# Evaluation of the suitability of free-energy minimization using nearest-neighbor energy parameters for RNA secondary structure prediction

**DOI:** 10.1186/1471-2105-5-105

**Published:** 2004-08-05

**Authors:** Kishore J Doshi, Jamie J Cannone, Christian W Cobaugh, Robin R Gutell

**Affiliations:** 1 The Institute for Cellular and Molecular Biology, The University of Texas at Austin, 1 University Station A4800, Austin, TX 78712-0159, USA

## Abstract

**Background:**

A detailed understanding of an RNA's correct secondary and tertiary structure is crucial to understanding its function and mechanism in the cell. Free energy minimization with energy parameters based on the nearest-neighbor model and comparative analysis are the primary methods for predicting an RNA's secondary structure from its sequence. Version 3.1 of Mfold has been available since 1999. This version contains an expanded sequence dependence of energy parameters and the ability to incorporate coaxial stacking into free energy calculations. We test Mfold 3.1 by performing the largest and most phylogenetically diverse comparison of rRNA and tRNA structures predicted by comparative analysis and Mfold, and we use the results of our tests on 16S and 23S rRNA sequences to assess the improvement between Mfold 2.3 and Mfold 3.1.

**Results:**

The average prediction accuracy for a 16S or 23S rRNA sequence with Mfold 3.1 is 41%, while the prediction accuracies for the majority of 16S and 23S rRNA structures tested are between 20% and 60%, with some having less than 20% prediction accuracy. The average prediction accuracy was 71% for 5S rRNA and 69% for tRNA. The majority of the 5S rRNA and tRNA sequences have prediction accuracies greater than 60%. The prediction accuracy of 16S rRNA base-pairs decreases exponentially as the number of nucleotides intervening between the 5' and 3' halves of the base-pair increases.

**Conclusion:**

Our analysis indicates that the current set of nearest-neighbor energy parameters in conjunction with the Mfold folding algorithm are unable to consistently and reliably predict an RNA's correct secondary structure. For 16S or 23S rRNA structure prediction, Mfold 3.1 offers little improvement over Mfold 2.3. However, the nearest-neighbor energy parameters do work well for shorter RNA sequences such as tRNA or 5S rRNA, or for larger rRNAs when the contact distance between the base-pairs is less than 100 nucleotides.

## Background

The biological functions of 16S, 23S and 5S rRNAs, tRNAs, telomerase RNA, Group I and II introns, RNaseP, and other structural RNAs are dictated by their three-dimensional structures. Thus, an accurate depiction of an RNA's secondary and tertiary structure is fundamental for our understanding of the mechanisms and consequences of its function, and an accurate prediction of an RNA folding into its secondary and tertiary structure from its primary structure will have a significant effect on our study of molecular biology. This RNA folding problem is usually divided into two components: the first is the determination of an RNA's folding pathway, and the second is the accurate and reliable prediction of an RNA's secondary and tertiary structure from its primary structure. In this paper, we focus on the second aspect and in particular RNA secondary structure prediction, which is a difficult problem. It has been estimated that the number of secondary structures models is greater than 1.8^*n*^, where *n *is the number of nucleotides (nt) in the sequence[1]. For example, *Saccharomyces cerevisiae *Phe-tRNA is only 76 nt in length and has an estimated 2.5 × 10^19 ^secondary structure models, while a larger RNA, such as the 16S rRNA from *Escherichia coli*, with 1542 nt, has an estimated total of 4.3 × 10^393 ^possible secondary structure models.

The most popular method for predicting RNA secondary structure from a single sequence is free energy minimization using a dynamic programming approach[2,3], based on energy parameters determined according to the nearest-neighbor model[4-10]. Programs based on this technique include Mfold[2,11], RNAStructure[12,13] and RNAFold[14]. Mfold is the most popular program in use today. By default, Mfold determines the optimal (minimum energy) structure and a set of suboptimal foldings that are within 12 kcal/mol (*default setting*) of the minimum energy structure. The set of suboptimal foldings exists and covers such a large energy range due to uncertainties in the thermodynamic data[2]. Mfold applies the following constraints: 1) only G:C, A:U and G:U base-pairs are formed (due to limitations of the energy parameters), 2) hairpin loops have at least three bases, and 3) no pseudoknotted structures are formed[15]. Attempts have been made to characterize the reliability of an RNA secondary structure prediction using dot plots[16].

Comparative analysis is another method for predicting RNA secondary and some tertiary structure[17-25]. Comparative analysis of RNA sequences and structures is a knowledge-based technique based on two fundamental assumptions: 1) different, homologous RNA sequences are capable of folding into the same secondary and tertiary structure, and 2) during the course of evolution, the secondary and tertiary structure of an RNA molecule remains mostly unchanged, while the primary structure can change significantly. The accuracy of the comparative method has recently been established using high-resolution crystal structures for the 30S and 50S ribosomal subunits[26,27]. Over 97% of the secondary structure base-pairs predicted by comparative analysis are present in the crystal structures[28].

The superior performance of the comparative method may lead one to incorrectly assume that the other methods for RNA secondary structure prediction are no longer necessary. Different methods for predicting RNA secondary structure are utilized in different situations and can have different objectives. Free energy minimization based RNA structure prediction methods are usually applied to a single RNA sequence. The most energetically stable RNA secondary structure(s) that are composed of canonical G:C, A:U, and G:U base-pairs and organized into standard helices are predicted. Non-canonical base-pairs and base-pairs not in standard helices cannot be predicted at this time. In contrast, RNA comparative sequence analysis methods predict a structure by searching an alignment for base-pairings that are common to all sequences in the dataset. This latter method can accurately predict canonical and non-canonical base-pairs that occur in secondary and tertiary structures. However, RNA comparative analysis is an iterative process requiring substantial sequence data, accurate sequence alignments, and the analysis of a structure that is common to all of the sequences in the dataset. In order to create an initial alignment, sequences must have significant identity to be aligned accurately while having sufficient variation (and covariation) to identify potential base-pairs and posit a structural hypothesis. The structural hypothesis is subsequently tested, refined, and expanded by the addition of more sequences to the alignment. Much of this process is still done manually, as computational tools to automate the process do not currently exist. The most recent comparative structure model for SSU rRNA is based on an alignment of 7,000 sequences[28]. The data was collected and the model was refined over a period of 20 years. The sequences included in this analysis are very diverse, spanning the entire tree of life.

In 1995 and 1996, the Gutell Lab conducted two comprehensive studies that: 1) determined how well the optimal secondary structure model predicted with the program Mfold (version 2.3) matched the structure model determined with comparative analysis for a set of 16S and 23S rRNAs, and 2) examined other aspects of the folding, such as the prediction accuracy of "short-range" base-pairs (base-pairings where the 5' and 3' halves of the base-pair are separated by 100 nt or less), or the prediction accuracy for base-pairs in different loop environments, to learn more about differences between the thermodynamic and comparative models[29,30]. The most significant findings from those studies were: 1) the average accuracy of the optimal prediction for a 16S rRNA was 46% while the average accuracy for a 23S rRNA was 44%. 2) The accuracies of the predicted secondary structure models for at least one individual 16S or 23S rRNA sequence were as high as 80% and as low as 10%. 3) On average, the Archaeal rRNAs were predicted with the highest accuracy, followed by the (eu)-Bacterial and Chloroplast, then Mitochondrial and Eukaryotic Nuclear rRNAs. 4) Short-range pairings, which comprised 75% of the total comparative base pairings for both 16S and 23S rRNA, were predicted more accurately than long-range pairings (base-pairings where the 5' and 3' halves of the base-pair are separated by more that 100 nt). 5) Base-pairs closing hairpin loops were predicted more accurately than those closing internal and multistem loops.

Since those studies were completed in 1995, four new developments have directly affected RNA structure prediction. 1) A new version of Mfold (3.1) was released with energy parameters revised to include sequence dependence and different secondary structure motifs[31]. 2) The accuracy of the comparative model for ribosomal RNA was established[28] with high-resolution crystal structure data from both the small and large ribosomal subunits[26,27]. 3) The number of available 16S and 23S rRNA secondary structures determined by comparative analysis increased significantly[24]. 4) Faster computers, which have significantly decreased the time it takes to fold an individual sequence, had become available to facilitate large-scale folding studies. These developments afforded us the opportunity to do a comprehensive re-evaluation of Mfold.

For more than 20 years, the basic paradigm for RNA secondary structure prediction, from a single sequence, has essentially remained the same: global free energy minimization with refinements to the nearest-neighbor energy parameters and minimization algorithms in an attempt to improve prediction accuracy. Refinements to the energy parameters, summarized in multiple sources[31-33], included measures for effects such as base-pair mismatches, base-pair positioning in helices, internal, bulge and multistem loops, and coaxial stacking. Newer versions of the program Mfold included these refinements in energy parameters in addition to improvements in the folding algorithm[31,34,35]. We questioned whether the improvements in the energy parameters and the algorithms could result in dramatic improvements in the accuracy and reliability of RNA secondary structure prediction programs such as Mfold, or would the energy-based RNA folding approach need to be fundamentally altered.

To begin to address this question, we analyzed the ability of Mfold 3.1 to predict RNA secondary structure models determined with comparative analysis. In addition, we compared the predictions and accuracies of Mfold 3.1 with its predecessor, Mfold 2.3, for a large set of phylogenetically diverse 16S and 23S rRNA sequences. All metrics considered in previous studies to evaluate the accuracy of Mfold 2.3[29,30] were revisited here. In addition, we analyzed the suboptimal population of predicted secondary structures and characterized metrics such as the number of suboptimals that were better (or worse) than the optimal structure prediction, the total number of comparative base-pairs observed, and the number of times a given base-pair is predicted in a set of optimal and suboptimal structure predictions. Only the most significant findings and metrics were discussed here; the reader is referred to the website[36] for a detailed presentation of all results from this analysis.

## Results

### Comparative structure database

The dataset assembled for this study was significantly larger than previous studies comparing RNA structure models predicted by comparative analysis and the Mfold folding program[29-31,35]. In particular, the 1995 and 1996 studies conducted by the Gutell Lab with Mfold 2.3 analyzed only 56 16S[29] and 72 23S[30] rRNA sequences respectively, and the 1999 study by Mathews *et al*. with Mfold 3.1 analyzed a total of 151,503 nt and 43,519 comparatively predicted canonical base-pairs (*i.e*., G:C, A:U and G:U) from 955 sequences, which included 22 16S, 5 23S, and 309 5S rRNA sequences, 484 tRNA sequences 23 Group I and three Group II intron sequences, 91 SRP sequences, and 16 RNase P sequences[31]. For this study, our sequence set included all three types of rRNA (5S, 16S and 23S) and Type I tRNA. As shown in Table 1, we analyzed a total of 1,411 RNA sequences, encompassing 1,505,143 nt and 385,854 canonical secondary structure base-pairs. Of the 1,411 sequences analyzed, 569 were tRNA, 496 were 16S rRNA, 256 were 23S rRNA, and 90 were 5S rRNA.

While the size of the comparative structure databases increased significantly between this study and previous Gutell Lab studies, only minor differences exist between the 1995 and 2004 versions of the 16S and 23S rRNA comparative structure models. For the 2004 version of the *Haloferax volcanii *16S rRNA secondary structure model, 30 base-pairs were added, 17 base-pairs were removed, and 427 base-pairs remained unchanged, resulting in a net difference of approximately 3% in the total number of base-pairs in the model. Similar numbers were observed for the other comparatively predicted structures evaluated. The comparative structure database used by Mathews *et al.*(1999) utilized known modified nucleotide information in tRNA to limit the base-pairing potential for those nucleotides that are modified[31]. In this study, rRNA or tRNA base modifications were not taken into account. A simple analysis of our tRNA dataset shows that 70% of our tRNA sequences came from genomic DNA sequences: as a result, no modification data was available for those sequences. For the remaining 30%, the number of modifications that could prevent A-form helix formation was minimal; between only 1 to 5 modifications per sequence.

Our dataset was extremely diverse in sequence and structure and included sequences from each of the three major phylogenetic domains, the Archaea, Bacteria, and Eucarya[37]. The eukaryotic dataset included sequences encoded in the Nucleus, Chloroplast and Mitochondrion. Since a dataset with sequences that were nearly identical would be less useful than a dataset with significant variation between the sequences, we characterized the diversity of the sequences in our dataset by calculating sequence identity for all pairs of 16S and 23S rRNA sequences within the different phylogenetic classifications of our dataset.

**Figure 1:**
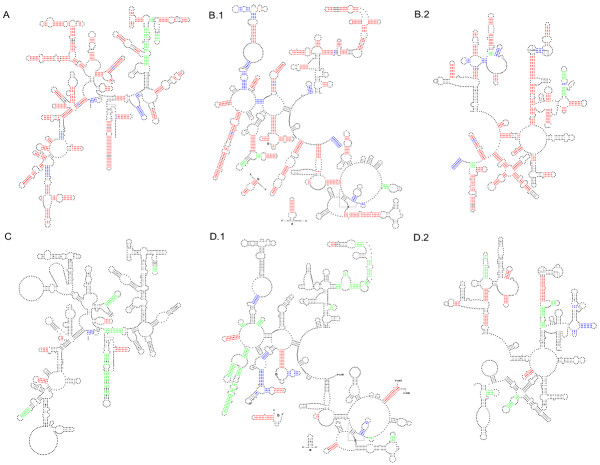
**Direct Comparison of Mfold 2.3 and Mfold 3.1 Folding Accuracy for Selected 16S and 23S rRNAs.** Base-pairs marked in red are predicted correctly by both Mfold 2.3 and Mfold 3.1. Base-pairs marked in blue are predicted correctly only by Mfold 2.3, and base-pairs marked in green are predicted correctly only by Mfold 3.1. Black base-pairs are not predicted correctly by either version of Mfold. Only canonical base-pairs in the comparative models in the current study and previous Gutell Lab studies are considered. Non-canonical base-pairs in the comparative structure models are not counted. Full-sized versions of each annotated structure diagram are available at our website[36]. **A: ***Archaea 16S rRNA Haloferax volcanii*. **B.1: ***Archaea 23S rRNA*, *5' half, Thermococcus celer*. **B.2: ***Archaea 23S rRNA, 3' half, Thermococcus celer*. **C: ***Eukaryotic Nuclear16S rRNA*, *Giardia intestinalis*. **D.1: ***Eukaryotic Nuclear 23S rRNA*, *5' half, Giardia intestinalis*. **D.2: ***Eukaryotic Nuclear 23S rRNA*, *3' half*, *Giardia intestinalis*.

For our 16S rRNA dataset, 75% of the Archaeal, 86% of the Bacteria, 71% of the Chloroplast, 99% of the Mitochondrial, and 94% of the Eukaryotic Nuclear sequence pairs had less than 80% sequence identity, while only 4% or fewer of the pairs in a given phylogenetic classification had 95% or more sequence identity (Table 1). Moreover, 79% of the Mitochondrial and 48% of the Eukaryotic Nuclear 16S rRNA sequence pairs had less than 50% sequence identity (Figure 1). The 23S rRNA dataset exhibited even more diversity than the 16S rRNA dataset, as 87% of the Archaeal, 94% of the Bacteria, 76% of the Chloroplast, 99% of the Mitochondrial, and 97% of the Eukaryotic Nuclear sequence pairs had less than 80% sequence identity, while 2% or fewer of the sequence pairs in a given phylogenetic classification had more than 95% sequence identity (Table 1). This demonstration of significant sequence variation between sequences in the same phylogenetic categories reveals the relative independence of the sequences within our dataset.

### RNA secondary structure prediction

The most important parameters used to control RNA secondary structure prediction by Mfold are window size (W), percent suboptimality (P), and the inclusion or exclusion of additional energy calculations based on coaxial stacking (efn2). The percent suboptimality variable establishes the energy range for computed foldings. The range is ΔG_min _to ΔG_min _+ ΔΔG, where ΔΔG is P% of ΔG_min_. The window size variable establishes the difference between the suboptimal folds by requiring that given folding has at least W base-pairs that are at least a distance W from any base-pairs in the foldings already computed. The program efn2 is used to re-compute the energetics of each predicted structure based on coaxial stacking opportunities with the structure. The structures are then re-ordered by the modified ΔG and a new optimal structure is selected. Previous studies by the Gutell Lab, Konings *et al*.[29] and Fields *et al*.[30], with Mfold 2.3 used window sizes of 10 and 20, respectively, with no efn2 re-evaluation; the selection of window size was limited by the computational resources available at the time the studies were conducted. Mathews *et al*.[31] used Mfold 3.1 with a window size of 0, percent suboptimality of 20%, and efn2 re-evaluation.

Each of the 1,411 sequences in our dataset was folded with Mfold 3.1, using a window size (W) of 1, percent suboptimality (P) of 5%, and maximum number of suboptimal foldings (MAX) of 750. The optimal, or minimum, free energy prediction and 749 suboptimal predictions were determined after re-ordering the structure predictions by the efn2 re-computed energetics. (**Note: **some sequences did not yield 749 suboptimal structure predictions under the folding conditions used in this study.) We configured the folding of our RNA sequences to: 1. maximize the number of structures predicted for any given sequence, 2. densely sample the suboptimal population close to the minimum free energy structure, since the structure with the lowest free energy (based on nearest-neighbor energetics) is expected to be most similar to the structure observed in nature for the free energy minimization techniques, and 3. to include coaxial stacking in the energy calculations with the efn2 option in Mfold 3.1.

The only difference between the run parameters from the previous Gutell Lab studies and this study was the significantly smaller window size used in the current study. The difference in window size affects the number of suboptimal structures computed. Since the previous Gutell Lab studies did not include any energy re-computation and re-ordering of predicted structures for potential coaxial stacking, this difference would not have a significant impact on the results.

The study by Mathews *et al*. used different values for percent suboptimality and window size in computing suboptimal structure predictions. The net result of the difference is that the Mathews *et al*. study considered suboptimal structures with energy values further away from the minimum free energy prediction than in our study. This difference could have an impact on the results since the Mathews *et al. *study may include a structure prediction for a given sequence that is not very energetically stable (and would be excluded from the suboptimal population in our study) but upon efn2 re-ordering becomes the minimum energy structure. If this predicted structure was more accurate than any other prediction in the population, the Mathews *et al. *study would reflect a higher prediction accuracy score for that given sequence.

### Overall accuracy for RNA structure prediction with Mfold 3.1

Given that 97–98% of the RNA secondary structure base-pairs predicted with comparative analysis were verified with the high resolution crystal structures[28], we scored the accuracy of the structures predicted with Mfold 3.1 by quantifying how well the optimal (minimum energy) structure prediction matched the comparative structure model for each sequence in our dataset. Results were only based on sequences folded in their entirety. We calculated accuracy by dividing the number of comparative base-pairs that were predicted exactly with Mfold by the total number of canonical base-pairs in the comparative model (excluding any base-pairs with IUPAC symbols other than G,C,A or U, see *Prediction Accuracy Calculations *in **Methods**). This method for calculating accuracy was the same as the previous Gutell Lab studies that utilized Mfold 2.3[29,30], with the exception that previous studies excluded comparative base-pairs that were pseudoknotted from consideration.

In contrast, base-pairs predicted with Mfold 3.1 in the Mathews *et al.*[31] study were considered correct if: 1. they matched a comparatively predicted base-pair exactly or 2. either nucleotide of the Mfold predicted base-pair (X,Y where X and Y are the positions of the nucleotides in the sequence) is within one nucleotide of its comparatively predicted position (X, Y ± 1 or X ± 1,Y). While the Mathews *et al. *study included a measure of the percentage of comparative base-pairs considered pseudoknotted, we were unsure if those base-pairs were specifically excluded from their accuracy calculations. Based on these differences in the accuracy calculations, the Mathews *et al. *study is reporting higher accuracies than our study.

Direct comparisons between the current study and the two previous Gutell Lab studies are meaningful due to the use of similar methodologies to calculate prediction accuracy. The only difference is the scoring method between the studies is the exclusion of pseudoknotted base-pairs from previous Gutell Lab studies. However, direct comparison of results between the current study and the Mathews *et al. *study are impacted by differences in the folding parameters and the scoring criteria.

#### Raw folding accuracy

The compilation of the accuracies for each sequence and the accuracy ranges for each RNA type and phylogenetic grouping were summarized in Table 2. The average folding accuracies per sequence for 5S rRNA and tRNA, the two smallest molecules in this study, were 71% and 69% respectively. The study by Mathews *et al. *reported average accuracy per sequence of 78% for 5S rRNA and 83% for tRNA[31]. Accuracies for our sets of 5S rRNAs and tRNAs were about 25% higher than the average accuracies for the 16S (41%) and 23S (41%) rRNAs. By comparison, the Gutell Lab's previous studies using Mfold 2.3 reported an average folding accuracy of 46% for 16S rRNA and 44% for 23S rRNA[29,30]. The study by Mathews *et al. *reported average accuracies (for folding complete RNA sequences) of 51% for a dataset of 22 16S rRNAs and 57% for a dataset of 5 23S rRNAs[31]. When considering only sequences analyzed in previous Gutell Lab studies, the average prediction accuracy with Mfold 3.1 was 45% for 16S rRNA and 43% for 23S rRNA (Table 2).

#### Variation in observed folding accuracy

To gauge the variation in accuracy score for the optimal structures predicted with Mfold, the percentages of scores greater than 60% and less than 20%, the median accuracy score, and the highest and lowest accuracy scores were identified for the four RNA types (Table 2). This analysis revealed a large range of accuracy scores with values significantly larger and smaller than the respective average value. For our current analysis, the highest accuracy score for the optimal structure for each RNA type was 100% for tRNA (*i.e., *at least one of the predicted tRNA structures had 100% of the base-pairs in the comparative model), 98% for 5S rRNA, 77% for 16S rRNA, and 74% for 23S rRNA. In contrast, at least one of the optimal folds for 5S rRNA or tRNA had a score of 0 (*i.e., *none of the base-pairs in the comparative structure model were predicted with Mfold). The lowest accuracy score was 5% for 16S rRNA and 1% for 23S rRNA.

The median accuracy score observed for each RNA type was 70% for tRNA, 81% for 5S rRNA, 41% for 16S rRNA and 41% for 23S rRNA. For 16S and 23S rRNA the overwhelming majority (86% for 16S rRNA and 89% for 23S rRNA) of optimal structures predicted with Mfold had an accuracy score greater than 20% and less than 60%, a trend also observed in our previous studies (Table 2)[29,30]. The majority of optimal structures predicted for 5S rRNA (77%) had an accuracy score greater than 60% (Table 2). For the tRNA, 60% of the optimal structures were predicted with accuracy greater than 60% and 39% of the optimal structures predicted with accuracy between 20% and 60%. The percentage of predicted structures with an accuracy score below 20% was highest for 23S rRNA (6%); increased from 1% previously[30], followed by 16S rRNA (4%); decreased from 9% previously[29], 5S rRNA (4%), and tRNA (2%) (Table 2). Our website[36] contains a complete list of accuracy scores and secondary structure diagrams indicating base-pairs that were predicted correctly for all sequences in our dataset.

#### Phylogenetic dependence in observed folding accuracy

Our previous thermodynamic-based folding analysis of 16S rRNAs[29] and 23S rRNAs[30] also revealed significant variation in the accuracy scores within and between the five major phylogenetic groups. For our current 16S rRNA dataset, the Archaeal sequences had the highest average accuracy (62%), while the Mitochondrial sequences had the lowest average accuracy (30%). Between these two extremes were the Bacteria (49%), Chloroplast (46%), and Eukaryotic Nuclear (34%) sequences (Table 3). These results were consistent with our previous studies, except that the Archaeal and Bacterial accuracy scores were slightly lower in our newer analysis (62% vs. 68% and 49% vs. 56%)[29]. For 23S rRNA (Table 3), the Archaeal dataset again had the highest accuracy scores (58%), followed by the Bacterial (49%), Eukaryotic Nuclear (42%), Chloroplast (39%), and Mitochondrion (30%). These results were also consistent with the trends observed in the previous studies, although the accuracy values for the current study were slightly less than the earlier analysis.

#### Direct comparison of structure predictions by Mfold 2.3 and Mfold 3.1 for specific RNA sequences

To access specific differences between the optimal foldings from Mfold 2.3 and Mfold 3.1 for select 16S and 23S rRNA sequences, we mapped the base-pairs predicted with both versions of Mfold onto the comparative structure models for each sequence. Some of the base-parings were predicted correctly with both versions of Mfold, other base-pairings were predicted exclusively by one version, while a third set of base-pairings were not predicted correctly with either version. The *Haloferax volcanii *16S rRNA (Figure 1A) and *Thermococcus celer *23S rRNA (Figure 1 B.1 and B.2) sequences were generally predicted very well with both versions of Mfold. Meanwhile, *Giardia intestinalis *16S (Figure 1C) and 23S (Figure 1 D.1 and D.2) rRNA sequences were predicted poorly with both versions of Mfold. The base-pairings in the comparative model that were missed by both versions of Mfold were generally longer range (see *Accuracy and the RNA Contact Distance*). This relationship between the comparative structure model for *G. intestinalis *16S and 23S rRNA and the poor prediction of this structure with both versions of Mfold (Figure 1C, D.1 and D.2) was representative of other sequences predicted with low accuracy by Mfold 2.3. A total of 9 out of 10 16S sequences and 7 of the 8 23S sequences predicted with accuracy of 30% or less with Mfold 2.3 were still predicted with less than 30% accuracy with Mfold 3.1 (Table 4).

Six significant results came from this section. 1) Direct comparison of previous Gutell Lab studies with the current study (in light of the significantly larger size and richness in sequence variation of the comparative structure database for the current study and the inclusion of comparative base-pairs in pseudoknots) suggests that refinements in the energy parameters and folding algorithm have not improved the accuracy of the Mfold program between versions 2.3 and 3.1 for the set of 16S and 23S rRNAs analyzed. 2) The discrepancies between the results of the Mathews *et al. *study and our study could be due to the different methods by which the sequences were folded and prediction accuracy calculated. 3) The accuracy scores for the majority of the 16S and 23S rRNA secondary structure models predicted with Mfold 2.3 and 3.1 were between 20% and 60%, while the accuracy scores for the majority of 5S secondary structure models predicted with Mfold were greater than 60%. 4) Some secondary structure models predicted with Mfold 2.3 and 3.1 have accuracy scores less than 20%. 5) The folding accuracy for Archaeal rRNAs was the highest, followed by Bacterial, Eukaryotic Chloroplast, Nuclear, and Mitochondrial rRNAs. 6) Sequences that were well-predicted with Mfold 2.3 tend to be well-predicted with Mfold 3.1, and sequences that were poorly-predicted using Mfold 2.3 tend to be poorly-predicted using Mfold 3.1.

### Accuracy and the RNA contact distance

For a given protein, the average sequence separation between pairs of amino acids involved in non-covalent interactions is defined as the "Contact Order"[38]. Two similar topological descriptions for non-covalent interactions in RNA are: 1) "RNA Contact Distance" is the separation on the RNA sequence between two nucleotides that base-pair, and 2) "RNA Contact Order" is the average of the RNA Contact Distances for a given RNA sequence. We considered any base-pair with a contact distance of 100 nt or less to be "short-range," a contact distance of 101–501 nt to be "mid-range," and a contact distance of 501 or greater to be "long-range." The majority of base-pairs in the 16S and 23S rRNA secondary structure models predicted with comparative analysis were short-range (Table 5), and previous studies have established that short-range base-pairs are predicted more accurately than long-range base-pairs[29,30]. In this section, we: 1) compared the accuracies of the short-range interactions predicted with Mfold 3.1 and Mfold 2.3, 2) compared the number of short-, mid-, and long-range base-pairs in the comparative models with those predicted by Mfold 3.1, and 3) determined the relationship between the base-pair prediction accuracy and the contact distance for 16S rRNA.

#### Accuracy of Short-range interactions

The 496 16S rRNA comparative structure models in this study were comprised of 191,994 canonical base-pairs. A total of 145,058 (76%) of these base-pairs were short-range, and 75,763 (52%) of these base-pairs were predicted correctly by Mfold 3.1 (Table 5). The average accuracy for short-range base-pairs was 50% per sequence (Table 5) (see *Per Sequence Averages *in **Methods **for a discussion on how per sequence averages are computed). By comparison, in the 1995 study, an average accuracy of approximately 55% per sequence was observed for short-range base-pairs[29].

For the 23S rRNA dataset, the 256 comparative structures contained a total of 178,958 canonical base-pairs. 134,085 (75%) of the 23S rRNA comparative, canonical base-pairs were short-range, and 67,130 (50%) of those base-pairs were predicted correctly by Mfold 3.1 (Table 5). The average prediction accuracy for short-range base-pairs was 47% per sequence (Table 5). In the 1995 study, an average accuracy of approximately 53% per sequence was observed for short-range base-pairs[30].

#### Distribution by RNA contact distance of comparative and Mfold predicted base-pairs

A total of 223,957 base-pairs were predicted with Mfold 3.1 for our 16S rRNA dataset (Table 5). This was 31,963 more than in the 16S rRNA comparative structure models (Table 5). Of the 223,957 base-pairs, 150,886 (67%) were short-range and 73,071 (33%) were mid- or long-range. Of the 150,886 short-range base-pairs, 75,763 (50%) were correct while only 6,171 (8%) of the mid- and long-range base-pairs were correct.

A total of 29,573 long-range base-pairs were predicted with Mfold 3.1, while the comparative models contained only a total of 3,932 long-range base-pairs; in other words, 13% of the total number of 16S rRNA base-pairs predicted with Mfold 3.1 were long-range while only 2% of the comparatively predicted base-pairs were long-range. Finally, of the 29,573 long-range base-pairs predicted by Mfold 3.1, only 193 (0.7%) were correct.

Similar results were observed for our 23S rRNA dataset (Table 5). A total of 218,908 base-pairs were predicted by Mfold 3.1; 137,780 (63%) were short-range, while 81,128 (37%) were mid- or long-range. 67,130 (49%) of the total short-range base-pairs predicted were correct, but only 10,758 (13%) of the total mid- and long-range base-pairs predicted were correct. Akin to the 16S rRNA dataset, a total of 36,989 (17%) 23S rRNA long-range base-pairs were predicted with Mfold 3.1, while only 7,752 (4%) of the comparatively predicted base-pairs were long-range. Only 1,317 of the 36,989 (4%) long-range base-pairs predicted by Mfold 3.1 were correct.

#### Relationship between prediction accuracy and RNA contact distance

**Figure 2:**
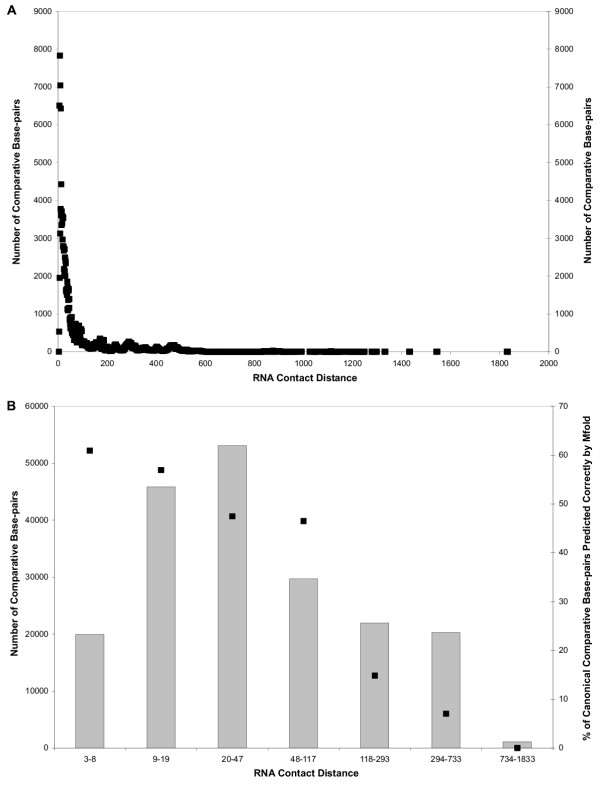
**Accuracy of Comparatively Predicted Base-pairs from 496 16S rRNA Sequences and RNA Contact Distance. A. **The RNA contact distance (the number of nucleotides in the RNA sequence that are separates the 5' and 3' base-paired) for all 191,994 base-pairs in comparative structure models is determined and plotted. **B. **The 191,994 comparatively predicted base-pairs are divided into seven RNA contact distance bins (see *Logarithmic Binning of Base-pairs by Contact Distance for 16S rRNA *in **Methods**) represented by columns. The accuracies for all base-pairs in each bin are also plotted as points.

These results prompted a more sophisticated analysis to quantify the relationship between the accuracy of base-pairs predicted with Mfold 3.1 and RNA contact distance. Figure 2A shows the distribution of contact distances for the 191,994 canonical base-pairs from the 496 16S rRNA comparative structure models in this study. The frequency of base-pairs observed decreases exponentially as contact distance increases. Based on this observation, we divided the 191,994 16S rRNA comparative base-pairs into seven somewhat equally-sized bins (within one order of magnitude of one another) by considering the contact distance values on a logarithmic scale instead of a linear scale. (see *Logarithmic Binning of Base-pairs by Contact Distance for 16S rRNA *in **Methods**).

The Mfold prediction accuracy for each of these bins was determined. The prediction accuracy was 61% for base-pairs in the smallest contact distance bin, 3–8, 57% for base-pairs in the 9–19 contact distance bin, 47% for base-pairs in the 20–47 bin, 46% for the 48–117 bin, 15% for the 118–293 bin, 7% for the 294–733 bin, and 0% for the 734–1833 bin (Figure 2B). The approximately linear relationship obtained from plotting the accuracy for logarithmically-scaled bins revealed an exponential relationship between the accuracy of Mfold and the contact distance (Figure 2B).

Five significant results were observed from the analysis in this section. 1) The accuracy of the predictions for short-range base-pairings was similar for Mfold 3.1 and Mfold 2.3. 2) More base-pairs were predicted with Mfold for any given sequence than in the corresponding comparative structure model. 3) Significantly more long-range base-pairs were predicted with Mfold 3.1 than in the comparative structure models. 4) The number of base-pairs in the comparative structure models decreases exponentially as the contact distance increases. 5) Base-pairs with a contact distance between 3 and 8 were predicted with the highest accuracy (61%) by Mfold, and accuracy values decreased exponentially as the RNA contact distance increases. The complete set of results for the prediction of 16S and 23S rRNA short-, mid-, and long-range base-pairs with Mfold 3.1 and comparisons with the comparative structure models are provided at our website[36].

### Suboptimal foldings

One of the features of the dynamic programming algorithm for free energy minimization employed by Mfold was the ability to provide a set of suboptimal structure predictions in addition to the minimum free energy or optimal structure prediction[2,12]. Mathews *et al. *included metrics which consider how suboptimal population may impact the prediction accuracy[31]. In this section, we introduce new metrics to continue the examination of the suboptimal population using the 496 16S rRNA sequences in our dataset. Due to the differences in the folding parameters and in the methods for computing accuracy, our survey of the suboptimal population should not be directly compared with the Mathews *et al. *study. Rather, our metrics provide a different perspective from which to excogitate the importance of the suboptimal structure predictions. In particular, we considered: 1) the amount of structural variation and the ΔΔG difference (before efn2 based re-evaluation) for pairs of structure predictions in the suboptimal population, 2) how many additional unique, canonical base-pairs in the comparative models were found in the suboptimal population, and how many incorrect base-pairs were predicted, and 3) which comparative base-pairs were predicted correctly in all, an intermediate number, or no structure predictions, in the set of suboptimal foldings.

#### Structural variation and ΔΔG difference for structure predictions in the suboptimal population

It has been previously noted that suboptimal structure predictions can be very similar or very different from one another[31]. Here we tested for a relationship between the ΔΔG (before efn2 based re-evaluation) and the structural variation score (see *Suboptimal Structural Variation Score *in **Methods**) for pairs of structure predictions within a suboptimal population. Higher structural variation scores indicate that two structures compared were more different from one another, while lower structural variation scores indicate that two structures were more similar. We analyzed the two 16S rRNA sequences with the highest and lowest optimal accuracy before efn2 re-evaluation and re-ordering in the Archaea dataset, *Haloferax volcanii *and *Methanospirillum hungatei*. The accuracy for *H. volcanii *based on the pre-efn2 minimum free energy structure prediction was 80%, while *M. hungatei *was predicted at 46% accuracy (Table 6). For the suboptimal population of each sequence, we calculated the structural variation and the difference in free energy for all possible pairwise comparisons. The total number of unique pairwise combinations for each sequence was 280,875, based on a total of 750 structure predictions (optimal plus 749 from the suboptimal population).

**Figure 3:**
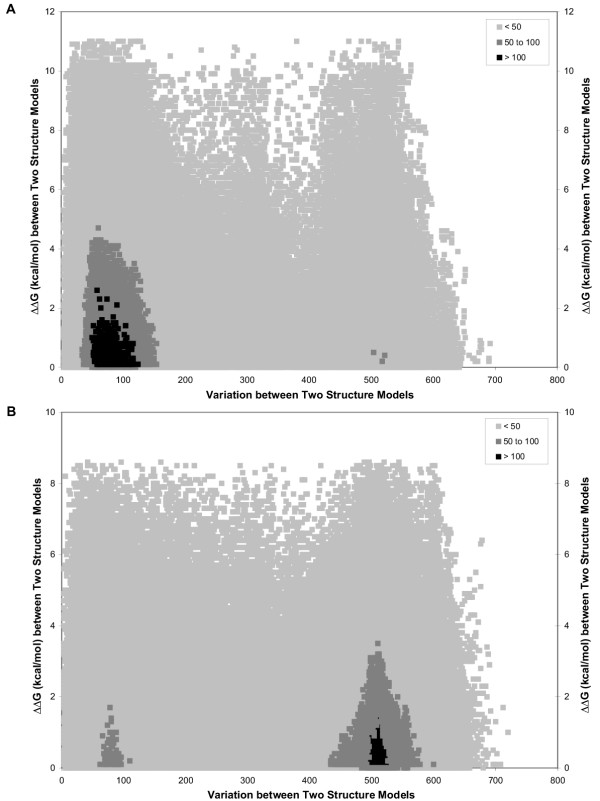
**ΔΔG vs. Structural Variation for Pairwise Comparisons from the "Suboptimal Population".** A set of 750 structure predictions (optimal + top 749 suboptimal) are compared, resulting in a total of 280,875 pairwise comparisons. The ΔΔG (pre efn2 re-evaluation) for two structure predictions is calculated by taking the absolute value of the difference between the ΔG of each structure prediction before efn2 re-evaluation. Structural variation for two structure predictions is calculated by counting the number of nucleotides in each structure prediction that either 1) have different pairing partners or 2) are paired in one structure prediction and unpaired in the other structure prediction (see *Suboptimal Structural Variation Score *in **Methods**). The shading within the figure indicates the number of pairwise comparisons that have the same values for both ΔΔG and structural variation score. **A: ***Archaea 16S rRNA Haloferax volcanii*. **B: ***Archaea 16S rRNA Methanospirillum Hungatei*.

For *H. volcanii*, 24,621 pairs (9%) of structure predictions had a structural variation score of 501 or higher, while 134,44 pairs (48%) had a score of 100 or less (Table 6). Thus, the majority of the structural predictions in the suboptimal population were more similar with one another. The observed ΔΔG range was the same for both categories, 0–11 kcal/mol (Table 6). More striking was the similarity in the average ΔΔG. For those pairwise comparisons with a structural variation score of 100 or less, the average ΔΔG was ~2.60 kcal/mol (weighted) (Table 6). For those pairwise comparisons with a structural variation score of 500 or higher, the average ΔΔG was 2.24 kcal/mol (Table 6). These results were summarized graphically in Figure 3A.

In contrast with *H. volcanii*, our analysis for *M. hungatei *revealed that a significant number of the structure predictions were different from one another. A total of 103,462 pairs (37%) of structure predictions had a structural variation score of 501 or higher, while only 43,376 pairs (16%) had a score of 100 or less (Table 6). The observed ΔΔG range was slightly smaller than in *H. volcanii*, 0–8.6 kcal/mol, while the average ΔΔG values were similar for *H. volcanii *and *M. hungatei*. For the pairwise comparisons with a structural variation score of 500 or higher, the average ΔΔG was 1.82 kcal/mol (Table 6). For those pairwise comparisons with a structural variation score of 100 or less, the average ΔΔG was ~2.05 kcal/mol (weighted) (Table 6). These results were summarized graphically in Figure 3B.

The most important observation from this section was that the ΔΔG between two structure predictions appeared to be independent of the similarity between the structure predictions. For example, *H. volcanii *pairwise comparisons with a structural variation score of 500 or higher had an average ΔΔG of 2.24 kcal/mol, while those pairwise comparisons where the score was 100 or less had an average ΔΔG of ~2.60 kcal/mol (weighted). In other words, free energy alone was not sufficient to adequately distinguish between different structure predictions. While the results suggest that suboptimal structural variation score could potentially be used as an indicator of the reliability of the structure prediction by Mfold, further investigation is required to evaluate the extent of this correlation.

#### Number of comparative base-pairs in the suboptimal population

For each individual 16S rRNA sequence, we identified the set of unique comparative base-pairs present from the collection of all base-pairs predicted in the suboptimal population. Since most comparative base-pairs were observed in more than one suboptimal structure, the total number of unique base-pairs observed was much lower than the total number of comparative base-pairs in the suboptimal population. Our entire 16S rRNA dataset of 496 comparative structure models contained a total of 191,994 unique canonical, comparative base-pairs (Table 7). 81,934 of these canonical base-pairs were predicted with Mfold 3.1 to be in a minimum free energy structure (after efn2 re-evaluation and re-ordering), an average accuracy of 41% per sequence (Table 7) (see *Per Sequence Average *in **Methods**). However, when considering the entire suboptimal population of structure predictions for each sequence, a total of 137,000 comparative canonical base-pairs were predicted correctly by Mfold, an average accuracy of 71% per sequence (Table 7). This represented a 30% increase in the average number of base-pairs in the comparative model that were predicted correctly per sequence. The average accuracy per sequence for an Archaeal, Bacterial, Eukaryotic Nuclear, Chloroplast, and Mitochondrial sequence increased by 21%-41% respectively (Table 7), and the largest increase for a single sequence (68%) was observed in the Mitochondrial dataset (Table 7).

However, these dramatic improvements in accuracy were offset by a significant increase in the number of base-pairs predicted incorrectly; Mfold experienced a large drop in selectivity. The total number of unique incorrect base-pair predictions for the 496 optimal structure predictions was only 142,023, while the total number of unique incorrect predictions was 2,372,305 for the 496 sets of optimal plus 749 suboptimal structure predictions, a 1,664% increase in the number of incorrect predictions (Table 7).

Two significant results came from this analysis. 1) When all of the base-pairs in the suboptimal population were included in the accuracy computation, we observed a 30% increase in average accuracy per sequence. 2) This same collection of suboptimal structures contained a 1,664% overall increase in the number of base-pairs that were not in the comparative model compared to the optimal structure prediction. In other words, a large decrease in selectivity was observed.

#### Distribution of base pairs throughout a suboptimal population

In the previous section, we considered the unique base-pairs predicted with Mfold 3.1. As mentioned earlier, the number of unique base-pairs is very small compared to the total number of base-pairs predicted within the top 750 predicted structures. A total of 166,690,139 base-pairs were predicted in the top 750 structure predictions for all 496 16S rRNA sequences in our dataset (some of the sequences did not yield 750 structure predictions with the Mfold folding parameters used in this study, see *Counts of Suboptimal Predictions More or Less Accurate than the Optimal Structure Prediction for Different 16S rRNA Sequences *under **Additional Information **at our website[36]). Of these, 59,454,137 were correct, while 107,236,002 were incorrect. In this section, we investigated 1) the frequency at which each base-pair in the comparative structure model appeared in the set of 750 structures (*i.e*., optimal + suboptimal population) predicted with Mfold 3.1 and 2) the relationship between this frequency and the RNA contact distance.

**Figure 4:**
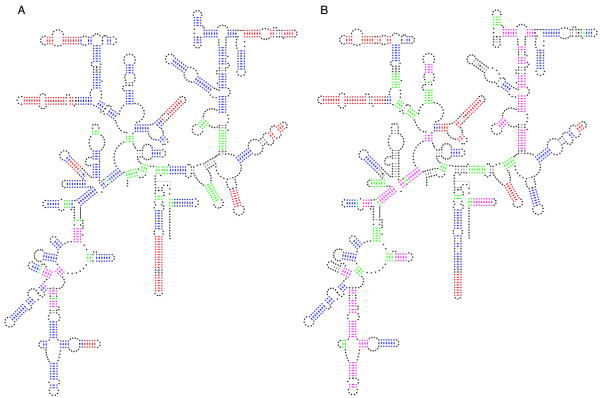
**Frequency of Base-pair predictions within a "Suboptimal Population" for selected 16S rRNAs.** The frequency of the prediction of each of the base-pairs in the comparative structure model in a set of 750 structure predictions (optimal + top 749 suboptimal) is displayed on the comparative structure model. Base-pairs marked in red are predicted correctly in all 750 structure predictions. Base-pairs marked in blue are predicted correctly in 600 to 749 structure predictions. Base-pairs marked in magenta are predicted correctly in 151 to 599 structure predictions, base-pairs marked in green are predicted correctly in only 1 to 150 structure predictions, and base-pairs marked in black are not predicted in any of the 750 structure predictions (some are non-canonical or occur in pseudo-knots, and thus are not expected to be predicted correctly). Full-sized versions of each annotated structure diagram are available at our website[36]. **A: ***Archaea 16S rRNA Haloferax volcanii*. **B: ***Archaea 16S rRNA Methanospirillum hungatei*.

The frequency of prediction with Mfold 3.1 for each base-pair in the comparative structure model was displayed for two Archaeal 16S rRNA comparative structure models in Figure 4. Given the analysis in the previous section on suboptimal structural variation, we selected *H. volcanii *and *M hungatei *for the panels in Figure 4. For both *H. volcanii *16S rRNA (Figure 4A), and *M. hungatei *16S rRNA (Figure 4B), some comparative base-pairs were predicted correctly in all 750 structure predictions, while others were predicted correctly in 600–749 structure predictions, 151–599 structure predictions, and 1–150 structure predictions. A few of the canonical base-pairs in the comparative structure model were not predicted in any of the 750 structure predictions. For *H.volcanii *(Figure 4A), the majority of the base-pairs predicted correctly were present in 600 to 749 structure predictions, with a significant number of base-pairs predicted correctly in all 750 structure predictions. Base-pairs predicted correctly in all 750 structure predictions were almost exclusively short-range (RNA contact distance less that 100 nt), while those predicted correctly in only 1–150 structure predictions were almost always long-range (RNA contact distance greater than 100 nt). This distribution was different for the *M. hungatei *16S rRNA (Figure 4B). Here, smaller numbers of base-pairs were predicted correctly in all 750 structure predictions, and more base-pairs were predicted correctly in only 151 to 599 of the structure predictions. Similar to *H. volcanii*, the majority of base-pairs predicted correctly in all 750 structure predictions have small RNA contact distances. For both sequences, short-range and long-range base-pairs were observed that were predicted correctly in zero structure predictions.

The distribution of comparatively predicted base-pairs, as observed from up to 750 structures of the suboptimal population, as a function of the RNA contact distance for all 496 16S rRNA sequences in our dataset, was summarized in Table 8. 76% of the base-pairs were short-range (RNA contact distance less than 101 nt), 22% were mid-range (RNA contact distance of 101–500 nt), and 2% were long-range (RNA contact distance greater than 500 nt). Of the comparative base-pairs predicted correctly in all 750 structure predictions for each 16S rRNA sequence in our data set, 98% were short-range, representing almost 15% (21,049 out of 137,000) of the total number of base-pairs predicted correctly. In contrast, only 2% of long-range base-pairs were predicted correctly in 600 or more structure predictions, representing <<1% (33 out of 137,000) of the total number of base-pairs predicted correctly. For comparative base-pairs predicted correctly in 600–749 structure predictions, 94% were short-range, for 151–599 structure predictions, 81% were short-range, and for 1–150 structure predictions, 70% were short-range. 80% (1,161 out of 1,460) of the long-range base-pairs predicted correctly appeared in 150 or fewer structure predictions. A total of 54,994 canonical, comparative base pairs were never predicted correctly, an average of 111 per 16S rRNA considered; 54% of these base pairs were short-range, 42% were mid-range, and 4% were long-range.

Three important observations were presented in this section. 1) For a given sequence, some of the comparative base-pairs were predicted correctly in all 750 structures (optimal + suboptimal population). 2) A sequence with higher optimal accuracy contained a larger percentage of the comparative base-pairs predicted correctly in more of the structure predictions within the suboptimal population, compared to a sequence with lower optimal accuracy. 3) Base-pairs predicted correctly in more suboptimal structure predictions tend to have a smaller RNA contact distance.

## Conclusions

In this paper, we evaluated how well the computer program Mfold 3.1[31], with the newest nearest-neighbor energy values, can predict the secondary structure base-pairs in comparative structure models for different RNAs. This study expands upon previous studies conducted by this lab in four ways. First, we analyzed 5S rRNA and tRNA sequences in addition to 16S and 23S rRNA sequences. Second, the number of comparative structure models in the current dataset was significantly larger, with a total of 1,411 RNAs (vs. 56 16S[29] and 72 23S[30] rRNAs studied previously), 1.5 million nucleotides and over 400,000 base-pairs, which covered all three phylogenetic domains and exhibited significant sequence variation (Table 1). Third, the increase in the speed of computers allowed us to analyze the best 749 suboptimal predictions in addition to the optimal prediction. Finally, the latest version of Mfold (version 3.1) was used. Our five most important conclusions are summarized hereunder.

1) *The comparative structure models for most sequences are predicted with similar accuracy by Mfold 2.3 and Mfold 3.1 *(Figure 1, Table 2,3,4) *when the differences between the datasets for previous Gutell Lab studies and the current study (e.g., sample size, minor differences in comparative models, sequence variation within the dataset) are considered. *The average folding accuracy for our current study is 41% for complete 16S rRNA sequences and 41% for complete 23S rRNA sequences, which is slightly less than in our earlier studies (Table 2). While the majority of optimal structure predictions for each RNA sequence still have an accuracy score between 20% and 60%, sequences with accuracy scores less than 20% are also observed (Table 2,4). On average, Archaeal and Bacterial 16S and 23S rRNA sequences still have higher accuracy scores than Eukaryotic Nuclear, Chloroplast, and Mitochondrial sequences (Table 3).

2) *Base-pairs with smaller RNA contact distances are both abundant in comparatively predicted structures and predicted more accurately by both versions of Mfold, and base-pairs with large RNA contact distances which are abundant in Mfold predicted structures only, are frequently incorrect. The prediction accuracy for individual base-pairs decreases exponentially as RNA contact distance increases. *Figure 2A shows that the number of comparative base-pairs decreases exponentially as the RNA contact distance increases. Using a logarithmic scale, we show that the base-pair prediction accuracy decreases in a linear fashion as contact distance increases (Figure 2B), which indicates an exponential relationship between base-pair prediction accuracy and RNA contact distance. In addition, many more long-range base-pairs (RNA contact distance of 501 or higher) are predicted than found in the corresponding comparative structure model, and the overwhelming majority of these predicted base-pairs are incorrect (Table 5).

3) *While uncertainties in the energy parameters may play a small role, free energy (calculated in its current form) alone is insufficient to distinguish between different structural possibilities for the same sequence. *The variation between any two structure predictions within the suboptimal population is not correlated with the ΔΔG between those two structure predictions (Table 6, Figure 3). We observe that two structures that are more similar with one another (structure variation score of less than 100), and very different from one another (structure variation score greater than 500), have very similar ΔΔG values.

4) *Without prior knowledge of the correct structure model, analysis of the suboptimal structure models can not improve our ability to both predict correctly the base-pairs in the secondary structure and assemble them into a single secondary structure model. *Our analysis of the accuracy of base-pairs predicted in the suboptimal population for our 16S rRNA dataset reveals that the entire population contained a higher percentage of base-pairs present in the comparative model than the optimal structure prediction alone (Table 7). When considering the suboptimal population, the free energy minimization method is able to identify an average of 71% or more of the comparatively predicted base-pairs for a given sequence (Table 7) vs. only 41% when considering just the optimal structure prediction. However, this same suboptimal population contains a significant increase in the number of incorrect base-pairs (Table 7). In other words, the increase in recall is offset by the inability of Mfold to consistently identify a single structure model containing a high percentage of comparative base-pairs.

5) *The frequency of correctly predicted base-pairs in the suboptimal population is extremely variable, and base-pairs with a smaller RNA contact distance are more likely to be observed at a higher frequency. *A qualitative analysis of Figure 4 shows that some base-pairs in the comparative structure model are predicted in all structure predictions within a suboptimal population, others are predicted in a subset, and yet others are not predicted at all. 98% of base-pairs predicted correctly in all structure predictions have an RNA contact distance less than 101 nt, while 80% of base-pairs with an RNA contact distance of 501 nt or more are only predicted correctly in 150 or less structure predictions (Table 8). Additionally, 63% (2,472 out of 3,932) of base-pairs with an RNA contact distance of 501 nt or more are never predicted correctly in the suboptimal population (Table 8).

From our previous analysis with version 2.3 of Mfold, we had determined that free energy minimization does not consistently identify the correct base-pairs in the 16S and 23S rRNA comparative secondary structure models[29,30]. We arrive at the same conclusion with our analysis of the current version 3.1 of Mfold and a significantly larger set of rRNA comparative structure models. One explanation could be incorrect energy parameters for multi-stem loops. Especially with longer sequences such as 16S or 23S rRNA, many long-range base-pairs occur along with the formation of these multi-stem loops. As we have shown in Table 5, only 8% (6,171 out of 73,071) of 16S rRNA and 13% (10,758 out of 81,128) 23S rRNA base-pairs predicted by Mfold with an RNA contact distance 101 or more nucleotides are correct. A more accurate characterization of the energetics of multi-stem loops may lead to significantly better prediction accuracies. Mathews *et al. *have started to address this issue using experimental studies[39,40] and known RNA secondary structures[31] to generate multi-stem loop initiation parameters that can be used in energetic calculations.

We believe that another potential reason for the inaccurate structures predicted with Mfold is that kinetics plays a role in RNA folding. Here, we suspect that nucleotide interactions with smaller contact distances will form more quickly, as suggested by Higgs[41], and will dominate the number of base-paired interactions formed. Presumably, these short-range interactions that form rapidly will be in equilibrium with other helices with a minimum contact distance (driven by nearest-neighbor energetics), and in the process, prevent energetically stable helices with larger contact distances from forming[41]. Several of our results support these ideas: 1) In the 16S and 23S rRNA comparative structure models, 75% of the predicted base-pairings have a contact distance of 100 or less; 2) Minimum free energy structures for 16S rRNA (as predicted by Mfold) have almost 10 times more long-range base-pairs than the comparative structure models (Table 5); 3) 92% (75,763 out of 81,934) and 86% (67,130 out of 77,888) of correctly predicted base-pairs have a contact distance of 100 or less for 16S and 23S rRNA (Table 5); 4) The higher prediction accuracy observed for short RNA molecules such as 5S rRNA or tRNA (Table 2).

Knowledge-based approaches that incorporate comparative analysis and high-resolution crystal structure data have been successful in the prediction of protein structures[42-44]. With the recent increase in the number of high-resolution crystal structures for different RNA molecules, it has been suggested that similar approaches could be utilized to predict RNA structure[45]. We envision a knowledge-based RNA folding algorithm with three fundamental facets: 1) a kinetic model of RNA folding that includes cooperative formation of short-range base-pairs and helices, 2) a thermodynamic component provided by the nearest-neighbor model applied locally within different parts of the sequence, and 3) relationships between RNA sequence and structure elements and observed structural biases, for example tetraloops[46], AA:AG motifs at the ends of helices[47], a bias for unpaired adenosines in the secondary structure model [48], and Lone Pair Triloops[49]. Use of sequence-structure relationships requires evaluation of known two- and three- dimensional RNA structures, hence the "knowledge-based" facet of the algorithm. Some researchers in the field have begun to adopt this approach. In Mfold 3.1, free-energy bonuses are applied to certain classes of hairpin loops and the energetic parameters for multi-branch loops were tuned using comparatively predicted structures[31]. Other researchers have developed RNA secondary structure prediction algorithms that combine energetics and comparative sequence analysis [50-52]. We believe that a tuned algorithm of the form just described has the potential to predict RNA secondary and eventually tertiary structure more accurately and reliably than methods currently available.

## Methods

### RNA secondary structure prediction

All 1,411 sequences in our dataset were folded using Mfold 3.1[31]. The optional parameters used for this study were window size (W) of 1, percent suboptimality (P) of 5% (default value), and maximum number of possible foldings (MAX) of 750. The efn2 program was used to re-compute the energetics for each predicted structure for a given sequence. The predicted structures were then ordered by the efn2 calculated free energies, and the minimum free energy structure reported was the lowest energy structure after efn2 re-evaluation. Only a single structure was selected as the minimum free energy structure, and we did not look for other foldings with the minimum free energy. The previous Gutell Lab studies[29,30] used window sizes (W) of 10 and 20 respectively and no efn2 re-evaluation. The Mathews *et al.*[31] study used a window size (W) of 0, percent suboptimality (P) of 20%, and efn2 re-evaluation.

### Prediction accuracy calculations

The accuracy of an Mfold prediction for a sequence was determined by: 1) counting the number of canonical base-pairs (excluding any base-pairs with IUPAC symbols other than G,C,A, or U) in the comparatively-derived secondary structure model that appeared in the Mfold 3.1 prediction, and 2) dividing that value by the total number of canonical base-pairs in the comparatively-derived model. Any canonical, pseudoknotted base-pairs in a comparatively derived secondary structure model were included in the total number of comparatively predicted base-pairs for the given model. For example, in the 16S rRNA from *Archaeoglobus fulgidus*, the Mfold 3.1 optimal structure prediction contained 256 of the 448 comparatively predicted canonical base pairings in the *A. fulgidus *comparative structure model; thus, the accuracy was 65%. Although the comparatively-derived models included non-canonical pairing predictions (*e.g., *U:U), these were not considered in the accuracy measure, since Mfold 3.1 does not predict non-canonical pairings.

### Comparative structure database

A total of 1,411 secondary structure models were determined with comparative analysis (Table 1) and used to benchmark the accuracy of Mfold 3.1. For our tRNA secondary structure models, 30% of the sequences contained at least one known modification that would prevent the nucleotide from participating in A-helix base-pairs. These modifications were not included as constraints for Mfold to use in the secondary structure prediction. 41% (349 out of 842) of the rRNA secondary structure models were currently available (as of January 2004) at The Comparative RNA Web (CRW) Site[24]. The other diagrams were not available at the CRW Site for the following reasons: 1) visual improvements were needed for the diagrams, 2) a small number of base-pairs needed to be changed in the structure models and 3) the sequence and/or the structure was not publicly available, pending submission of a manuscript. The secondary structure drawing program XRNA[53], on Sun Microsystems computers, was used to draw the comparative structure diagrams.

### Per sequence averages

Some average values for statistics computed in this study, such as secondary structure prediction accuracy, were calculated on a per sequence basis. A *per sequence average *variant of a particular statistic was calculated by averaging the value of the statistic for each individual sequence in the dataset. For example, for the 16S rRNA dataset, the overall accuracy was calculated by first determining the accuracy of the Mfold optimal structure prediction for each individual sequence[36]. Then, the 496 accuracy values were averaged to calculate the overall accuracy score of 41%.

### Computational setup

All 1,411 sequences were folded on a computer with dual AMD Athlon MP 1800 processors and 1GB of RAM, under the SuSE Linux 8.0 operating system[54]. In addition, four single-processor AMD Athlon computers (Thunderbird 1GHz processor), each with 512 MB of RAM and SuSE Linux 8.0[54], were used to prepare sequences for folding and to compress the results for storage. The sequences were folded in under 48 hours using a workflow-based system that was developed for automatically managing the folding runs. Even after compression, the aggregate set of folding results required over 150 GB of disk space. The raw results were parsed and imported into a database, managed by MySQL[55]. The database contained over 100 tables that held intermediate results from the folding runs. Intermediate results were then retrieved, using simple SQL queries, when required to calculate final results.

### Logarithmic binning of base-pairs by contact distance for 16S rRNA

Figure 2A showed that the number of comparative base-pairs observed decreased exponentially as the contact increased; therefore, logarithmic binning was required to group the base-pairs into somewhat equally-sized bins based on contact distance. The shortest and longest contact distances observed in our 16S rRNA data set were 3 and 1833, respectively[36]. Therefore, the overall range of our logarithmic scale was from log_10 _(3) to log_10 _(1833). This range was divided into equal increments to define our contact distance bins. After evaluating many increment sets, with the requirement that the sizes of the bins be within one order of magnitude of one another, seven distance bins were established (Figure 2B).

### Suboptimal structural variation score

The suboptimal structural variation score measures the agreement between two different secondary structure models for the same RNA sequence. We compute the score by comparing the paired or unpaired state of each nucleotide in the two structure models. We increment the score when either a given position is unpaired in one structure model and paired in the other or when the position is paired to different positions in the respective structure models. We do not increment the score when a given position is paired to the same position in both structure models or is unpaired in both structure models. The higher the structural variation score, the lower the level of agreement between the two secondary structure models. The structural variation score is zero for two identical structure models. For two structure models that are different at every position the structural variation score equals the number of nucleotides in the sequence.

## Authors' contributions

KJD developed the workflow based management system for folding the sequences, constructed the database for analyzing the folding results, tabulated the results and drafted the manuscript. JJC developed the secondary structure models for the tRNA dataset, contributed ideas to the analysis and the figure and table generation, and edited the manuscript. CWC assembled the tRNA alignment, tabulated results, and edited the manuscript. RRG provided vision and direction and rewrote portions of the manuscript. All authors read and approved the final manuscript.

## List of abbreviations

Nucleotide – nt

## Figures and Tables

**Table 1:** Distribution of Comparatively Predicted Secondary Structure Models Analyzed

	5S rRNA	16S rRNA	23S rRNA	tRNA	Total
Structures	90	496	256	569	1,411
Total AGCU Nucleotides^1^	10,777	724,475	712,575	42,283	1,490,110
Total Nucleotides^2^	10,819	736,412	714,723	43,189	1,505,143
Total Comparative Pairings^3^	3,107	191,994	178,958	11,796	385,854
Average Sequence Length	120	1,485	2,792	76	-
Average Pairings/Structure^3^	35	387	699	21	-
*Phylogenetic Distribution*					
Archaea	12	23	17	76	128
Bacteria	28	195	75	155	453
Eucarya					
Nuclear	45	133	52	207	437
Chloroplast	4	33	31	131	199
Mitochondrion	1	112	81	-	194

*Pairwise Sequence Identity^4^*	Archaea	Bacteria	Nuclear	Chloroplast	Mitochondrion
16S rRNA					
< 80% Identity	380 / 75%	32,456 / 86%	16,574 / 94%	746 / 71%	12,332 / 99%
< 50% Identity	0 / 0%	0 / 0%	8,500 / 48%	98 / 9%	9,852 / 79%
>= 95% Identity	16 / 3%	1,588 / 4%	212 / 1%	4 / 0.4%	12 / 0.1%
Total Pairs	506	37,830	17,556	1,056	12,432
23S rRNA					
< 80% Identity	236 / 87%	5,214 / 94%	2,568 / 97%	710 / 76%	6,394 / 99%
< 50% Identity	0 / 0%	0 / 0%	1,960 / 74%	62 / 7%	5,830 / 90%
>= 95% Identity	6 / 2%	42 / 1%	8 / 0.30%	18 / 2%	8 / 0.1%
Total Pairs	272	5,550	2,652	930	6,480

*tRNA Amino Acid Distribution^5^*	Archaea	Bacteria	Nuclear	Chloroplast	Total
Alanine (Aln)	13	14	6	5	38
Arginine (Arg)	4	9	17	12	42
Asparagine (Asn)	3	9	10	3	25
Aspartic acid (Asp)	4	4	12	6	26
Cysteine (Cys)	2	5	3	5	15
Glutamine (Gln)	3	6	13	4	26
Glutamic acid (Glu)	4	8	23	8	43
Glycine (Gly)	6	18	17	9	50
Histidine (His)	3	6	10	7	26
Isoleucine (Ile)	3	16	8	10	37
Leucine (Leu)	-	-	-	-	-
Lysine (Lys)	3	8	15	4	30
Methionine (Met)	4	7	7	9	27
Phenylalanine (Phe)	3	6	16	10	35
Proline (Pro)	5	10	12	8	35
Serine (Ser)	-	-	-	-	-
Threonine (Thr)	7	14	6	10	37
Tryptophan (Trp)	1	6	3	10	20
Tyrosine (Tyr)	2	-	6	3	11
Valine (Val)	6	9	23	8	46
*Total*	76	155	207	131	569

^1 ^Considers only A, G, C or U nucleotides.

^2 ^Considers all nucleotides.

^3 ^Includes only G:C, A:U and G:U base-pairings predicted with comparative analysis.

^4 ^Average sequence identities for all pairwise comparisons between sequences. Number of pairwise comparisons equals (n^2^-n) where n is the number of sequences considered.

^5 ^Only Type I tRNAs are considered.

**Table 2:** Average Accuracy of the Optimal RNA Structure Predicted with Mfold 3.1^†^

	5S rRNA		16S rRNA			23S rRNA			tRNA	
	M^1^	C^2^	P_1_^3^	M	C	P_2_^4^	M	C	M^5^	C

Sequences	309	90	56	22	496	72	5	256	484	569
Accuracy^6,7,8,9^	78 ± 23	71 ± 24	46 ± 17	51 ± 16	41 ± 13 **45 ± 16**	44 ± 11	57 ± 14	41 ± 13 **43 ± 12**	83 ± 22	69 ± 24
High/Low^10^		98/0	81/10		77/5	74/19		74/1		100/0
Median		81			41			41		70
Distributions										
≤ 20% acc^11^		4	9		4	1		6		2
≥ 60% acc^12^		77	25		9	6		5		60
20%<acc<60%^13^		19	66		86	93		89		39

^†^All values are percentages unless otherwise indicated. All averages are *per sequence *averages for folding complete sequences as defined in the *Per Sequence Averages *section in **Methods**. C, Current Study; P_1_, Previous Study by Gutell Lab for 16S rRNA[29]; P_2_, Previous Study by Gutell Lab for 23S rRNA[30]; M, Previous Study by *Mathews et al.*[31]. Accuracies from all previous studies are for folding complete sequences.

^1 ^All sequences from the Mathews *et al. *study (M) were folded with Mfold 3.1 using a window size (W) of 0, percent suboptimality (P) of 20%, maximum number of suboptimals (MAX) of 750 and efn2 re-evaluation and re-ordering.

^2 ^All sequences in the current study (C) were folded with Mfold 3.1 using a window size (W) of 1, percent suboptimality (P) of 5% and efn2 re-evaluation and re-ordering

^3 ^All sequences in the previous Gutell Lab study on 16S rRNA (P_1_) were folded with Mfold 2.3 using a window size (W) of 10 and no efn2 re-evaluation and re-ordering.

^4 ^All sequences in the previous Gutell Lab study on 23S rRNA (P_2_) were folded with Mfold 2.3 using a window size (W) of 20 and no efn2 re-evaluation and re-ordering.

^5 ^Bases modified in tRNA that are subsequently unable to fit into an A form helix were constrained to be single-stranded.

^6 ^Comparative base-pairs that are pseudoknotted were excluded from the analysis in previous Gutell Lab studies (P_1_, P_2_), but were included in the current study. The Mathews *et al. *study included a measure of the percentage of pseudoknotted base-pairs in comparatively predicted structures they considered, but it was unclear if they were included in the analysis.

^7 ^In all studies, only canonical, comparative base-pairs (excluding any base-pairs with IUPAC symbols) were considered. For both the current study (C) and previous Gutell Lab studies (P_1_, P_2_), a predicted base-pair was considered correct only if it matched a comparative base-pair exactly. In the Mathews *et al. *(M) study, a base-pair was considered if: 1. it matched a comparatively predicted base-pair exactly or 2. either nucleotide of the Mfold predicted base-pair (X,Y where X and Y are the positions of the nucleotides in the sequence) is within one nucleotide of its comparatively predicted position (X, Y ± 1 or X ± 1,Y).

^8 ^Accuracy values in bold under the (C) columns for 16S and 23S rRNA represent average prediction accuracies in the current study for just the subset of sequences considered in the previous Gutell Lab studies.[29, 30]. The following sequences were considered in previous Gutell Lab studies, but excluded from the current study, *Olisthodiscus luteus *(16S rRNA, Chloroplast) and *Sulfolobus solfataricus *(23S rRNA, Archaea).

^9 ^When the efn2 re-evaluation and re-ordering step was omitted from our study, the average prediction accuracy was 40 ± 13 for 16S rRNA, 40 ± 13 for 23S rRNA, 69 ± 24 for 5S rRNA, and 66 ± 24 for tRNA. For complete details, see our website[36].

^10 ^Accuracy scores for the best and worst predicted structures in each group.

^11 ^Percentage of predicted structures with an accuracy of 20% or less.

^12 ^Percentage of predicted structures with an accuracy of 60% or higher.

^13 ^Percentage of predicted structures with an accuracy between 20% and 60%.

**Table 3:** Average Accuracy of the Optimal RNA Structure Predicted with Mfold 3.1 Grouped by Phylogeny^†^

	5S rRNA	16S rRNA		23S rRNA		tRNA
	C	P_1_	C	P_2_	C	C

Archaea	79 / 98 / 29	68 / 81 / 55	62 / 77 / 51	59 / 74 / 51	58 / 74 / 40	73 / 100 / 32
Bacteria	62 / 94 / 18	56 / 69 / 39	49 / 68 / 21	53 / 66 / 45	49 / 66 / 31	74 / 100 / 0
Eucarya (n)^1^	75 / 94 / 0	30 / 47 / 10	34 / 50 / 15	41 / 60 / 23	42 / 63 / 21	61 / 100 / 0
Eucarya (c)	67 / 85 / 16	48 / 71 / 32	46 / 71 / 19	39 / 54 / 19	39 / 49 / 21	73 / 100 / 19
Eucarya (m)		31 / 56 / 17	30 / 60 / 5	38 / 57 / 24	30 / 61 / 1	
Eucarya (m)^2^			31 / 60 / 5			
Eucarya (m)^3^			33 / 60 / 16			

^†^All values (average/high/low) shown as percentages unless otherwise indicated. The determination of the accuracy for the structures predicted with Mfold is described in the **Methods **section, *RNA Secondary Structure Prediction *and *Prediction Accuracy Calculations*. C, Current Study; P_1_, Previous study by the Gutell Lab for 16S rRNA[29]; P_2_, Previous study by the Gutell Lab for 23S rRNA[30].

^1 ^(n), Nuclear-encoded sequences; (c), Chloroplast-encoded sequences; (m), Mitochondrial-encoded sequences.

^2 ^Based on comparative models with 100 or more canonical base-pairs only.

^3 ^Based on comparative models with 300 or more canonical base-pairs only.

**Table 4:** RNA Folding Accuracy of Specific 16S and 23S rRNA Sequences using Mfold 2.3 and 3.1^†^

	Previous[29, 30]	Current
**16S rRNA**		
*Eukaryotic Mitochondrion*		
*Zea mays *(X00794)	17	30
*Ascaris summ *(X54253)	17	13
*Caenorhabditis elegans *(X54252)	23	24
*Eukaryotic Nuclear*		
*Hexamita sp. *(Z17224)	27	29
*Giardia muris *(X65063)	22	29
*Giardia ardeae *(G17210)	30	33
*Giardia intestinalis *(X52949)	10	23
*Encephalitozoon cuniculi *(X98467)	18	21
*Vairimorpha necatrix *(Y00266, M24612)	28	25
*Babesia bigemina *(X59064)	20	19
**23S rRNA**		
*Eukaryotic Chloroplast*		
*Astasia longa *(X14386)	19	23
*Eukaryotic Mitochondrion*		
*C. elegans *(X54252)	30	31
*Gallus gallus *(X52392)	28	25
*Saccharomyces. cerevisiae *(J01527)	27	20
*Z. mays *(K01868)	24	29
*Eukaryotic Nuclear*		
*E. gracilis *(X53361)	23	21
*G. intestinalis *(X52949)	24	33

^†^All values are percentages unless otherwise indicated. The determination of the accuracy for the structures predicted with Mfold is described in the **Methods **section, *RNA Secondary Structure Prediction *and *Prediction Accuracy Calculations*. Genbank accession numbers are listed in parentheses for each sequence.

**Table 5:** Accuracy of Base-pairs Predicted with Mfold 3.1 as a Function of RNA Contact Distance^†^

	16S rRNA			23S rRNA		
RNA Contact Distance	496 Structures			256 Structures		

*Comparative*	Total Base-pairs	% of Total		Total Base-pairs	% of Total	

Total	191,994	100		178,958	100	
2–100	145,058	76		134,085	75	
*2–50*	*121,170*	*63*		*106,534*	*60*	
*51–100*	*23,888*	*12*		*27,551*	*15*	
101+	46,936	24		44,873	25	
*101–500*	*43,004*	*22*		*37,121*	*21*	
*501+*	*3,932*	*2*		*7,752*	*4*	
*Predicted with Mfold 3.1*						
Total	223,957	100		218,908	100	
2–100	150,886	67		137,780	63	
*2–50*	*123,708*	*55*		*109,078*	*50*	
*51–100*	*27,178*	*12*		*28,702*	*13*	
101+	73,071	33		81,128	37	
*101–500*	*43,498*	*19*		*44,139*	*20*	
*501+*	*29,573*	*13*		*36,989*	*17*	
*Correctly Predicted with Mfold 3.1*		%C^1^	%M		%C	%M
Total	81,934	43	37	77,888	44	36
2–100	75,763	52	50	67,130	50	49
*2–50*	*64,651*	*53*	*52*	*54,898*	*52*	*50*
*51–100*	*11,202*	*47*	*41*	*12,232*	*44*	*43*
101+	6,171	13	8	10,758	24	13
*101–500*	*5,978*	*14*	*14*	*9,441*	*25*	*21*
*501+*	*193*	*5*	*0.7*	*1,317*	*17*	*4*
*Avg. Percent Correct^2^*	Current	Previous[29]		Current	Previous[30]	
2–100	50	55		47	53	
*2–50*	*52*	-		*49*	-	
*51–100*	*44*	-		*40*	-	
101–200	22	15		26	35	
201–300	10	14		22	21	
301–400	9	13		13	10	
401–500	4	12		16	13	
501+	4	-		14	-	

^†^All base-pairs predicted in the comparative and the Mfold optimal structure predictions including those base-pairs predicted correctly (any base-pairs with IUPAC symbols other than A,G,C, or U are excluded) are grouped by RNA contact distance for 16S and 23S rRNA. RNA contact distance is defined as the number of nucleotides intervening between the 5' and 3' halves of a base-pair. The determination of the accuracy for the structures predicted with Mfold is described in the **Methods **section, *RNA Secondary Structure Prediction *and *Prediction Accuracy Calculations*.

^1 ^%C, the percentage of comparatively predicted base-pairs; %M, the percentage of Mfold predicted base-pairs.

^2 ^The *Per Sequence Average *(see *Per Sequence Average *in **Methods**) percentage of comparative base-pairs in each distance category predicted correctly in the Mfold optimal structure predictions.

**Table 6:** Average, Minimum and Maximum ΔΔG Values for Pairwise Comparisons of Different Suboptimal Folds^†^

	*Haloferax volcanii*		*Methanosprillum hungatei*	
Optimal Accuracy^1^	80%		46%	
Total Fold Predictions (Optimal + Suboptimal)	750		750	
Total Pairwise Comparisons	280,875		280,875	
*Structural variation score of 1 to 50*				
Num of Pairwise Comparisons	32,378	12%^2^	11,016	4%
ΔΔG Min (kcal/mol)	0		0	
ΔΔG Max (kcal/mol)	11		8.60	
ΔΔG Average (kcal/mol)	2.84		2.19	
*Structural variation score of 51 to 100*				
Num of Pairwise Comparisons	102,071	36%	32,360	12%
ΔΔG Min (kcal/mol)	0		0	
ΔΔG Max (kcal/mol)	11		8.60	
ΔΔG Average (kcal/mol)	2.53		2.01	
*Structural variation score of 101 to 500*				
Num of Pairwise Comparisons	121,805	43%	134,037	48%
ΔΔG Min (kcal/mol)	0		0	
ΔΔG Max (kcal/mol)	11		8.6	
ΔΔG Average (kcal/mol)	2.24		1.74	
*Structural variation score of 501+*				
Num of Pairwise Comparisons	24,621	9%	103,462	37%
ΔΔG Min (kcal/mol)	0		0	
ΔΔG Max (kcal/mol)	11		8.6	
ΔΔG Avg (kcal/mol)	2.24		1.82	

^†^Both sequences are 16S rRNAs. For each sequence, Mfold 3.1 predicts one optimal or minimum free energy fold and 749 suboptimal folds (750 total folds). Pairwise comparisons are grouped based on the structural variation between the two folds compared. For details on how structural variation between two folds is calculated see Materials and Methods. The range of ΔΔG values observed is 0–11 kcal/mol for *H. volcanii *and 0–8.60 kcal/mol for *M. hungatei*., and all ΔG values are pre-efn2 re-evaluation.

^1 ^Without efn2 re-evaluation and re-ordering of predicted folds.

^2 ^Percentage of total pairwise comparisons.

**Table 7:** Distribution of 16S rRNA Base-pairs Predicted Correctly and Incorrectly^†^

	Overall	Archaea	Bacteria	Eucarya		
				
				(C)^1^	(M)	(N)
Comparative	191,994	10,211	83,385	13,406	29,979	55,013
Opt Correct^2^	81,934	6,376	41,032	6,105	9,459	18,962
Subopt Correct^3^	137,000	8,570	65,177	10,032	21,201	32,020
Opt Incorrect^2^	142,023	4,758	49,563	8,603	27,617	51,482
Subopt Incorrect^3^	2,372,305	101,253	947,197	161,397	472,614	689,844
Opt Accuracy^2,4^	41%	62%	49%	46%	30%	34%
Subopt Accuracy^3,4^	71%	84%	78%	75%	71%	59%
Avg Improvement^5^	30%	21%	29%	30%	41%	24%
Best Prediction^6^	92%	91%	89%	92%	92%	90%
Max Improvement^7^	68%	35%	54%	53%	68%	48%
Min Improvement^8^	10%	10%	12%	12%	14%	11%

^†^All 496 16S rRNA sequences are considered. Each sequence is folded for a population of one optimal and 749 suboptimal structure predictions. The determination of the accuracy for the structures predicted with Mfold is described in the **Methods **section, *RNA Secondary Structure Prediction *and *Prediction Accuracy Calculations*. Values are calculated by summing the number of unique base-pairs encountered for each sequence that satisfy each particular category (any base-pairs involving IUPAC symbols other than A,G,C, or U are excluded). For example, *Subopt Correct *is calculated by summing the number of unique, correctly predicted base-pairs encountered in the population of optimal plus suboptimal structure predictions for each of the 496 16S rRNA sequences. Prediction accuracy when including base-pairs predicted correctly in suboptimal structure predictions is also tabulated.

^1 ^(c), Chloroplast-encoded sequences; (m), Mitochondrial-encoded sequences; (n), Nuclear-encoded sequences.

^2 ^Considering only the optimal prediction.

^3 ^Considering the optimal prediction plus up to 749 suboptimal predictions.

^4 ^Averages calculated on per sequence basis. Please see *Per Sequence Averages *in **Methods**.

^5 ^Average improvement in Mfold secondary structure prediction accuracy when pooling base-pairs from both the optimal prediction and suboptimal predictions.

^6 ^The highest Mfold secondary structure prediction accuracy for an individual sequence when pooling base-pairs from both the optimal and suboptimal populations.

^7 ^The largest improvement in Mfold secondary structure prediction accuracy for an individual sequence when pooling base-pairs from both the optimal and suboptimal populations.

^8 ^The smallest improvement in Mfold secondary structure prediction accuracy for an individual sequence when pooling base-pairs from both the optimal and suboptimal populations.

**Table 8:** Frequency of Comparative Base-pairs in 750 Structures Predicted with Mfold 3.1^†^

	RNA Contact Distance									
Frequency^1^	2–100 nt			101–500 nt			501+ nt			

750	21,049	*98%*	**18%**	417	*2%*	**2%**	0	*0%*	**0%**	21,466
600–749	42,362	*94%*	**37%**	2,805	*6%*	**14%**	33	*0%*	**2%**	45,200
151–599	20,775	*81%*	**18%**	4,594	*18%*	**23%**	266	*1%*	**18%**	25,635
1–150	31,285	*70%*	**27%**	12,253	*27%*	**61%**	1,161	*3%*	**80%**	44,699
Correct	115,471	*84%*		20,069	*15%*		1,460	*1%*		137,000
Never	29,587	*54%*		22,935	*42%*		2,472	*4%*		54,994
Total	145,058	*76%*		43,004	*22%*		3,932	*2%*		191,994

^†^For all 496 16S rRNA sequences, a total of 750 structure models are predicted for each sequence (one optimal and 749 suboptimal structure predictions). Every base-pair (excluding any base-pairs involving IUPAC symbols other than A,G,C, or U) in the comparative structure model that appears in a set of 750 structure predictions for a particular sequence is categorized by 1) the number of structure predictions in which it appears and 2) the RNA contact distance. The four bold percentages for each of the three RNA contact distances each total 100%, and reveal the percentage of base-pairs predicted correctly for the four frequency ranges. For example, a total of 115,471 base-pairs with an RNA contact distance of 2–100 nt were predicted correctly. Of those base-pairs, 18% (21,049) were predicted in 750 structure predictions, 37% (42,362) were predicted in 600–749 structure predictions, 18% (20,775) were predicted in 151–599 structure predictions, and 27% (31,285) were predicted in 1–150 structure predictions. In contrast, the three italicized percentages for each of the four frequency ranges, and the "Correct", "Never", and "Total" categories total 100%. For example, 54,994 base-pairs were never predicted in 750 structure predictions. Of those base-pairs, 54% (29,587) have an RNA contact distance of 2–100 nt, 42% (22,935) have an RNA contact distance of 101–500 nt, and 4% (2,472) have an RNA contact distance of 501+ nt.

^1 ^Frequency of prediction throughout a suboptimal population of up to 750 structure predictions.
